# Bullying behavior and employee well-being: how do different forms of social support buffer against depression, anxiety and exhaustion?

**DOI:** 10.1007/s00420-022-01844-w

**Published:** 2022-03-11

**Authors:** Birgit Pauksztat, Denise Salin, Momoko Kitada

**Affiliations:** 1 Department of Business Studies, Uppsala University, Uppsala, Sweden; 2 Department of Management and Organization, Hanken School of Economics, Helsinki, Finland; 3 World Maritime University, Malmö, Sweden

**Keywords:** Workplace bullying, Social support, Mental health, Exhaustion, Maritime industry

## Abstract

**Purpose:**

Workplace bullying has severe negative consequences for the well-being of targeted employees. Previous research suggests that social support may buffer against such adverse effects. However, it remains unclear if different forms of support have equally strong effects and if support buffers equally effectively against different outcomes. Further, little is known about social support as a mitigating factor in remote occupational groups such as seafarers. This study examines the buffering effects of four forms of support (instrumental and emotional peer support, company support, non-work support) on three aspects of employee well-being (depression, anxiety, and exhaustion) among seafarers.

**Methods:**

Responses to a cross-sectional online survey from a convenience sample of 414 seafarers on international commercial vessels were analyzed using moderated regression analyses with PROCESS.

**Results:**

Exposure to workplace bullying behavior increased seafarers’ depression, anxiety and exhaustion. Instrumental peer support and non-work support buffered the negative impact of bullying on depression. The impact of bullying on exhaustion was buffered by company support. The impact of bullying on anxiety was not buffered by any of the four forms of support.

**Conclusion:**

Extending previous research, the findings suggest that the interaction between workplace bullying and support depends not only on the source of support, but also on the type of support and the outcome considered. While support from colleagues on board was important for seafarers, company and non-work support must not be overlooked. Interventions should, therefore, encourage the development of peer support and ensure access to shore-based support for workers in remote locations.

## Introduction

Involving “harassing, offending, or socially excluding someone or negatively affecting someone’s work” (Einarsen et al. 2020, p. 26), workplace bullying is typically characterized by repeated and prolonged exposure to predominantly psychological mistreatment (Einarsen et al. 2020; Nielsen and Einarsen 2018). Workplace bullying has severe negative effects for targeted employees (Boudrias et al. 2021; Mikkelsen et al. 2020; Nielsen and Einarsen 2012), and may be one of the greatest occupational risk factors for mental health problems (Schütte et al. 2014), increasing the risk of anxiety, depression, sleeping problems, and burnout (Boudrias et al. 2021; Mikkelsen et al. 2020; Nielsen and Einarsen 2012). Knowledge about factors that may mitigate or buffer the negative impact of workplace bullying on employees’ well-being is limited (Nielsen and Einarsen 2018). While existing research provides some evidence that contextual and organizational factors such as social support can have a buffering effect (Blomberg and Rosander 2020; Nielsen et al. 2020a), not all findings are consistent. Moreover, samples in previous studies predominantly included office and service workers; little is known about social support as a mitigating factor in remote occupational groups such as seafarers.

This study provides deeper insight into the buffering function of social support among seafarers on international commercial vessels. More specifically, the study extends existing research by comparing four forms of support—instrumental peer support, emotional peer support, company support, and non-work support from family and friends—and their buffering effects on three aspects of employee well-being: depression, anxiety and exhaustion. For seafarers, a remote occupational group at high risk of bullying, exhaustion and mental health problems (Jepsen et al. 2017; Sampson and Ellis 2019), a better understanding of the effects of different forms of social support is especially important for identifying effective ways of reducing the negative impact of bullying.

Social support involves the provision of “emotional, informational, or instrumental resources in response to the perception that others are in need of such aid” (Cohen et al. 2000, p. 4). Support may range from being instrumental and very tangible, such as providing practical assistance, to being emotional and more intangible, primarily focusing on boosting the recipient’s mood and morale (Cohen et al. 2000). Moreover, employees can get support from different sources, such as colleagues or family members.

For seafarers, relevant forms of support can be distinguished along two dimensions. The first dimension, reflecting the contrast made by seafarers between “ship” (i.e. those onboard their vessel) and “shore” (i.e. those ashore), is the distinction between onboard and shore-based sources of support. The second dimension is the distinction between instrumental and emotional support. This classification provides four forms of support that are the focus of this study (Table 1). Onboard the vessel, fellow crew members can provide two kinds of support: *instrumental peer support* (e.g. protecting targets, helping targets to get outside support) and *emotional peer support* (e.g. listening, showing empathy). In line with the ship/shore distinction, “fellow crew members” or “peers” here includes everyone on board, regardless of department or rank. From ashore, seafarers may receive company support and non-work support. *Company support*, i.e. support from the company’s shore personnel, is primarily instrumental as seafarers contact shore personnel for support with practical matters, typically related to personnel matters such as contracts, training or workload. *Non-work support* from family and friends is primarily emotional, as they are physically distant and do not have the means to provide tangible support. Non-work support largely depends on seafarers’ access to affordable, fast and reliable Internet or other means of communication. Of over 1500 seafarers surveyed in 2016, only half had access to Internet on board (Sampson et al. 2018).

**Table 1:** Four forms of support

		Type of support	
		Instrumental	Emotional
Location of source of support	Onboard	Instrumental peer support	Emotional peer support
	Ashore	Company support	Non-work support

Social support has been argued to have a protective effect by buffering the negative impact of demands and stressful events (Cohen and Wills 1985), such as bullying behavior. Previous research suggests several possible explanations for this buffering effect. First, the transactional theory of stress holds that stress arises when employees feel they cannot cope with the challenges they are faced with (Lazarus and Folkman 1984). This appraisal is not only related to the stressor itself, but also to the perceived ability to cope with it. Hence the availability of coping resources such as social support becomes crucial in determining how employees respond to potentially stressful situations such as bullying. This resonates with the core ideas of the Job Demands-Resources (JD-R) model (Bakker and Demerouti 2007), which posits that the strain experienced by employees can be explained by an interplay between demands and available resources. When faced with a severe stressor like bullying or other forms of mistreatment, social support may provide a contextual resource that helps employees to cope. Further, in line with Janoff-Bulman’s (1992) schema theory of trauma, traumatic experiences such as exposure to bullying can shatter targets’ basic cognitive schemas about the world as benevolent and fair, and their self-perceptions as worthy human beings able to control what happens to them (Mikkelsen et al. 2020). From this perspective, receiving social support should help to boost and restore targets’ view of the world and themselves. Finally, exposure to bullying may lead to changes in targets’ health behavior (Salin 2013), such as an increase in alcohol and drug consumption (Nielsen et al. 2020b), which in turn affect physical and mental health. Social support can encourage a healthy lifestyle (Callaghan and Morrissey 1993), thus reducing the chances of resorting to unhealthy coping strategies and helping targets to maintain good physical and mental health.

In general, based on the arguments provided above, all forms of support would generally be expected to reduce the adverse effects of bullying behavior. Indeed, existing research provides some evidence for the assertion that different forms of social support can act as a buffer (Blomberg and Rosander 2020; Gardner et al. 2013; Nielsen et al. 2020a; Warszewska-Makuch et al. 2015).

However, not all studies found significant interactions between workplace bullying and social support (e.g. Dehue et al. 2012). Even when the interaction effect was significant, the nature of the interaction differed between studies. Some studies found that social support was effective at buffering low levels of bullying, but was less effective at high levels of bullying (Blomberg and Rosander 2020; Warszewska-Makuch et al. 2015). Others found that the buffering effect of social support was stronger at high levels of bullying (Nielsen et al. 2020a), or that support exacerbated (rather than alleviated) the negative effect at high levels of mistreatment, i.e. a reverse-buffering effect (Lim and Lee 2011). The picture is further obscured by the fact that many studies merged different forms of social support into one measure (Dehue et al. 2012) or used outcome measures combining different aspects of well-being (Nielsen et al. 2020a; Blomberg and Rosander 2020; Warszewska-Makuch et al. 2015). Further, when different forms of support were considered, they were usually analyzed in separate models (i.e. one at a time), rather than tested directly against each other in the same model (for exceptions, see Blomberg and Rosander 2020; Gardner et al. 2013). Consequently, while it seems safe to conclude that social support matters, our knowledge of how social support buffers the impact of bullying on employee well-being is still limited.

Possible explanations for these contradictory results may be that researchers have focused on different aspects of well-being and on different forms of social support, both with regard to the sources of support and the types of support (i.e. instrumental vs. emotional). For instance, Warszewska-Makuch et al. (2015) found a significant interaction effect of coworker support but not of supervisor support on well-being, suggesting that the strength of the effect might differ for different forms of support. Lim and Lee (2011) reported that family support moderated the effect of incivility on depression but not on anxiety, which suggests that effects might differ for different outcomes. This also relates to a key notion in social support research, namely that different forms of support protect against different types of occupational stressors (Cohen and Wills 1985). Extending this reasoning, according to the triple-match principle (De Jonge and Dormann 2006), the buffering effect will depend not only on the type of the demand and the resource, but also on the outcome considered. That is, some forms of support may be more effective than others in buffering the negative impact of bullying behavior, and their effectiveness may differ for different outcomes.

In a seafaring context, the remote and isolated nature of the work may also affect the effectiveness of different forms of support. On the one hand, shore-based support may be difficult to access and, given the physical distance, may perhaps be less effective than onboard support. On the other hand, as crew members may have to live and work together in confined spaces over weeks or months, effective peer support may not be forthcoming. Crew members may be reluctant to provide support if they fear that their involvement might contribute to the escalation of the situation and/or lead to negative consequences for themselves (Kitada 2010; see also Blomberg and Rosander 2020).

Taken together, this raises questions about the role of different forms of support. It remains unclear if different forms of support have equally strong effects, and if support buffers equally effectively for different aspects of well-being. To examine this in more detail, we put forward the following research questions:

- **Research question 1** Do different forms of social support vary in their effectiveness to buffer the negative impacts of exposure to bullying behavior?
- **Research question 2** Does the buffering effect of social support vary for depression, anxiety, and exhaustion?

## Methods

### Study design and sample

The data for this study were collected between 25 July and 25 September 2020 through a cross-sectional online survey of seafarers. We used a convenience sampling approach to reach seafarers on international commercial vessels, distributing the survey as widely as possible online (i.e. via e-mail, websites and social media) through shipping companies, national and international industry organizations, maritime education institutions and welfare organizations. Participants received no financial compensation for their participation in the study.

The study is part of a research project “Global seafarers during the COVID-19 pandemic: Crew resilience, support and seafarers’ wellbeing”, which was approved by the World Maritime University's Research Ethics Committee (REC-20-27R). Data were collected in line with the 1964 Declaration of Helsinki. Participation was voluntary and participants remained anonymous. To start the survey, respondents provided informed consent by confirming that they were 18 or older, had read the information about the study and agreed to participate.

The analyses in this study are based on responses from seafarers who, at the time of the survey, had been on board for at least one week. Excluding respondents who in response to filter questions indicated that they did not meet these criteria, as well as respondents with missing values on one or more of the variables in the regression analyses left 414 respondents for the analyses in this paper.

Most of the 414 respondents were men (96%; information missing for 3 respondents). Respondents were between 19 and 65 years old (*M* = 40.4, SD = 10.4; information missing for 34 respondents), and had worked at sea between 0 and 47 years (*M* = 18.3, SD = 10.8). Most of the respondents (79%) were officers. At the time of the survey, respondents had been on board between less than a month and eighteen months (*M* = 4.1, SD = 3.8).

### Measures

Data were collected using a comprehensive questionnaire, and only a subset of the scales included in the questionnaire was used for the analyses in this study. Depression, anxiety, exhaustion and exposure to bullying behavior during the last seven days were measured using 5-point frequency scales, with answer categories 1 = “never”, 2 = “once”, 3 = “several times”, 4 = “almost every day” and 5 = “every day”. A seven-day period was chosen to broaden the range of potential respondents, as it allowed us to include seafarers with short work periods and those whose work period had just started. To measure instrumental peer support, emotional peer support and company support, respondents were asked to indicate the extent to which they agreed or disagreed with each statement on a 7-point scale, from 1 = “strongly disagree” to 7 = “strongly agree”.

Depression was measured based on the two-item Patient Health Questionnaire (PHQ-2) scale (Kroenke et al. 2003), i.e. “feeling sad, depressed or hopeless” and “My days have been filled with things that interest me” (reverse-coded). Cronbach’s *α* was 0.65.

Anxiety was measured based on the two-item General Anxiety Disorder (GAD-2) scale (Kroenke et al. 2007). A sample item is “feeling afraid, anxious or worried”. Cronbach’s *α* was 0.86.

Exhaustion was measured with three items from the seafarers’ exhaustion scale (Pauksztat 2017). A sample item is “feeling very tired during work”. Cronbach’s *α* was 0.83.

Exposure to bullying behavior was measured using the Short Negative Acts Questionnaire (S-NAQ; Notelaers et al. 2019). Respondents were asked to indicate how often they had been exposed to nine types of bullying behavior (e.g. “Insulting or offensive remarks about you as a person, your attitudes or your private life”) from “other crew members on this ship” during the last seven days.^1^ Cronbach’s *α* was 0.90.

Instrumental peer support was measured with two items adapted from Van Yperen and Hagedoorn (2003) regarding the availability of instrumental support from fellow crew members on board (e.g. “When I need help from other crew members, I get it”). Cronbach’s *α* was 0.82.

Emotional peer support was measured with four items developed for this survey. The items reflect different dimensions of interpersonal emotion management (Little et al. 2012), adapted to a seafaring context. A sample item is “When someone on this ship is sad, worried or in a bad mood, other crew members cheer him/her up.” Cronbach’s *α* was 0.93.

Company support was measured with three items based on the first author’s interviews with seafarers (unpublished data). The items were introduced by asking respondents to think about their experience when contacting the shipping company, i.e. the company’s shore personnel. A sample item is “I get the information or help that I need”. Cronbach’s *α* was 0.88.

Non-work support from family and friends at home during the last seven days was measured with two items written for this study. The items addressed two types of communication, i.e. communication “in writing, e.g. via email, sms or other text based chat services” and communication “by talking to them so that you could hear and/or see them (e.g. telephone, WhatsApp, Imo, Skype, …)”. Answer categories were 1 = “never”, 2 = “once”, 3 = “2–3 times”, 4 = “4–6 times”, 5 = “once every day”, and 6 = “several times per day”. Cronbach’s α was 0.59.

We collected information about respondents’ gender, age (in years), experience at sea (in years), hierarchical level (from 0 = “cadet” to 5 = “captain”), the expected length of their time on board according to their contract (from 1 = “about 2 weeks or less” to 7 = “9 months or more”), and the actual number of months they had been on board. Crew size was measured with one item, from 1 = “less than 5” to 9 = “more than 500”. To measure the workload on board during the last seven days, respondents rated the crew’s workload and their own workload, respectively, on a scale from 1 = “extremely low (‘holiday’)” to 7 = “extremely high” (*α* = 0.88).

### Statistical analyses

To assess our measurement model, we conducted a confirmatory factor analysis using Mplus version 8.4 (Muthén and Muthén 1998–2019), with robust maximum likelihood (MLR) and full information maximum likelihood (FIML) imputation. Control variables and non-work support (a formative measure) were not included. The confirmatory factor analysis indicated a good fit of the measurement model (*χ*^*2*^(254) = 482.24; RMSEA = 0.05, 90% confidence interval (CI) [0.04–0.05]; CFI = 0.95; SRMR = 0.05). All factor loadings were significant (*p* < 0.001) and exceeded the recommended value of 0.5 (Hair et al. 2019). Using Satorra and Bentler’s (2010) chi-square difference test for MLR, we found that the model fit the data significantly better than alternative models, including a five factor model with mental health (depression and anxiety), exhaustion, exposure to bullying behavior, peer support (instrumental and emotional) and company support (Δ*χ*^*2*^(11) = 160.35, *p* < 0.001; *χ*^*2*^(265) = 701.50; RMSEA = 0.06; CFI = 0.91; SRMR = 0.07), a three factor model with the dependent variables, exposure to bullying behavior and support variables (Δ*χ*^*2*^(18) = 548.87, *p* < 0.001; *χ*^*2*^(272) = 1193.79; RMSEA = 0.09; CFI = 0.81; SRMR = 0.09), and a one factor model (Δ*χ*^*2*^(21) = 6927.17, *p* < 0.001; *χ*^*2*^(275) = 2471.48; RMSEA = 0.14; CFI = 0.54; SRMR = 0.12).

To test our hypotheses, we used OLS regression with PROCESS version 3.5 (Hayes 2018) in SPSS version 27, with depression, anxiety and exhaustion as dependent variables. Models included the control variables, main effects and the interaction terms. To facilitate comparison, we included the same control variables in all models, selecting variables identified as predictors of mental health and/or exhaustion in previous studies on seafarers (Jepsen et al. 2017; Lefkowitz and Slade 2019; Oldenburg et al. 2013). Because of the small number of women in the sample (*n* = 15), we did not control for gender. As age and experience at sea were highly correlated (*r* = 0.906, *p* < 0.001), and because the high number of missing cases for age would have reduced the sample size to 380, we used experience at sea rather than age.

We conducted initial analyses where we added each interaction term separately (i.e. one at a time). In the final models, all four interaction terms were added simultaneously to assess their relative strength. Because PROCESS does not allow to include more than two interaction terms at a time, we first ran each model in SPSS. We then reran the model in PROCESS, specifying the two interaction terms with the highest *t* values as moderators, and including the other two interaction terms as covariates. Before creating the interaction terms, the antecedent variables were centered. Because initial SPSS analyses indicated the presence of outliers (standardized residuals greater than 3) for depression and anxiety as dependent variables, we used bootstrapping (bootstrap sample size = 50,000) to generate estimates of the means, standard errors and 95% percentile bootstrap confidence intervals.

## Results

Descriptive statistics and correlations are presented in Table 2. Exposure to bullying behavior had significant positive correlations with depression (*r* = 0.460, *p* < 0.001), anxiety (*r* = 0.417, *p* < 0.001) and exhaustion (*r* = 0.463, *p* < 0.001). Moreover, all four types of support had negative correlations with depression, anxiety and exhaustion; only the correlation between non-work support and anxiety was non-significant.

**Table 2:** Means, standard deviations and Pearson correlations, based on data from 414 respondents

		*M*	SD	1	2	3	4	5	6
1	Depression	2.52	1.01						
2	Anxiety	2.64	1.16	0.665***					
3	Exhaustion	2.61	1.03	0.727***	0.679***				
4	Exposure to bullying behavior	1.43	0.63	0.460***	0.417***	0.463***			
5	Instrumental peer support	5.67	1.11	− 0.445***	− 0.347***	− 0.411***	− 0.471***		
6	Emotional peer support	5.36	1.12	− 0.478***	− 0.275***	− 0.397***	− 0.359***	0.580***	
7	Company support	5.06	1.49	− 0.446***	− 0.378***	− 0.457***	− 0.381***	0.450***	0.477***
8	Non-work support	4.56	1.26	− 0.177***	− 0.084	− 0.134**	− 0.146**	0.200***	0.249***
9	Experience at sea	18.32	10.83	− 0.097*	− 0.101*	− 0.128**	− 0.142**	0.131**	0.097*
10	Hierarchical level	3.19	1.42	− 0.029	0.034	− 0.030	− 0.163**	0.069	0.023
11	Expected length of time on board	4.73	1.42	0.129**	0.135**	0.140**	0.133**	− 0.076	0.050
12	Months on board	4.10	3.79	0.252***	0.224***	0.287***	0.236***	− 0.172***	− 0.056
13	Workload	4.73	0.97	0.205***	0.232***	0.272***	0.123*	− 0.130**	− 0.128**
14	Crew size	4.29	1.16	0.126*	0.101*	0.118*	0.039	− 0.066	− 0.095

		7	8	9	10	11	12	13
8	Non-work support	0.231***						
9	Experience at sea	0.134**	0.029					
10	Hierarchical level	0.050	− 0.004	0.567***				
11	Expected length of time on board	− 0.053	0.159**	− 0.156**	− 0.281***			
12	Months on board	− 0.154**	0.122*	− 0.131**	− 0.189***	0.532***		
13	Workload	− 0.102*	− 0.106*	0.036	0.104*	0.007	− 0.019	
14	Crew size	− 0.173***	− 0.021	− 0.085	− 0.096	0.066	0.040	− 0.048

*p < 0.05, **p < 0.01, ***p < 0.001

Table 3 shows the regression results. Taken together, the variables explained a total of 42.3% of the variance in depression (*R*^2^ = 0.423), 31.7% of the variance in anxiety (*R*^2^ = 0.317), and 43.4% of the variance in exhaustion (*R*^2^ = 0.434). As would be expected, exposure to bullying behavior made a significant contribution to the variance in depression (*b* = 0.493, SE = 0.086, *p* < 0.001), anxiety (*b* = 0.617, SE = 0.099, *p* < 0.001) and exhaustion (*b* = 0.562, SE = 0.087, *p* < 0.001). Other stressors, notably workload and the number of months on board, significantly contributed to the variance in all three outcomes. In addition, hierarchical level contributed to the variance in anxiety, with those at higher hierarchical levels more frequently reporting symptoms of anxiety (*b* = 0.144, SE = 0.047, *p* < 0.01).

**Table 3:** OLS regression results for depression, anxiety and exhaustion as dependent variables

	Depression				Anxiety				Exhaustion			
	b	SE	LL	UL	b	SE	LL	UL	b	SE	LL	UL
Intercept	1.439	0.301	0.845	2.022	1.002	0.397	0.225	1.790	1.225	0.313	0.612	1.841
Experience at sea	0.000	0.004	− 0.009	0.008	− 0.010	0.006	− 0.021	0.001	− 0.005	0.005	− 0.014	0.004
Hierarchical level	0.046	0.035	− 0.021	0.115	0.144**	0.047	0.052	0.237	0.059	0.036	− 0.011	0.130
Expected length of time on board	0.022	0.031	− 0.040	0.082	0.040	0.041	− 0.042	0.120	− 0.002	0.034	− 0.070	0.062
Months on board	0.038**	0.012	0.014	0.062	0.035*	0.016	0.004	0.067	0.050***	0.013	0.026	0.076
Workload	0.108*	0.043	0.024	0.193	0.178***	0.052	0.075	0.281	0.201***	0.043	0.116	0.285
Crew size	0.050	0.034	− 0.016	0.117	0.053	0.041	− 0.028	0.134	0.045	0.035	− 0.021	0.116
Exposure to bullying behavior	0.493***	0.086	0.324	0.661	0.617***	0.099	0.422	0.811	0.562***	0.087	0.396	0.738
Instrumental peer support	− 0.075	0.050	− 0.172	0.025	− 0.096	0.068	− 0.228	0.041	− 0.070	0.053	− 0.172	0.039
Emotional peer support	− 0.242***	0.047	− 0.332	− 0.148	− 0.028	0.057	− 0.139	0.083	− 0.150**	0.049	− 0.248	− 0.056
Company support	− 0.108**	0.033	− 0.172	− 0.044	− 0.150***	0.041	− 0.230	− 0.070	− 0.154***	0.034	− 0.221	− 0.086
Non-work support	− 0.041	0.032	− 0.105	0.021	0.007	0.040	− 0.073	0.085	− 0.012	0.033	− 0.077	0.052
Interactions												
Exposure to bullying behavior * Instrumental peer support	0.137*	0.068	0.005	0.274	0.131	0.094	− 0.033	0.337	0.080	0.071	− 0.066	0.216
Exposure to bullying behavior * Emotional peer support	− 0.014	0.064	− 0.140	0.114	− 0.084	0.090	− 0.267	0.091	− 0.004	0.072	− 0.137	0.146
Exposure to bullying behavior * Company support	0.067	0.052	− 0.033	0.172	0.117	0.067	− 0.018	0.247	0.124*	0.058	0.001	0.231
Exposure to bullying behavior * Non-work support	− 0.144**	0.049	− 0.244	− 0.052	− 0.115	0.059	− 0.236	− 0.005	− 0.074	0.054	− 0.184	0.027
*R*^2^	0.423				0.317				0.434			
*F*	19.459				12.334				20.323			

Unstandardized coefficients and standard errors, with 95% confidence intervals, based on data from 414 respondents. Estimates based on bootstrapping (bootstrap sample size = 50,000)

LL = 95% confidence interval, lower limit. UL = 95% confidence interval, upper limit

*p < 0.05, **p < 0.01, ***p < 0.001

**Fig. 1:**
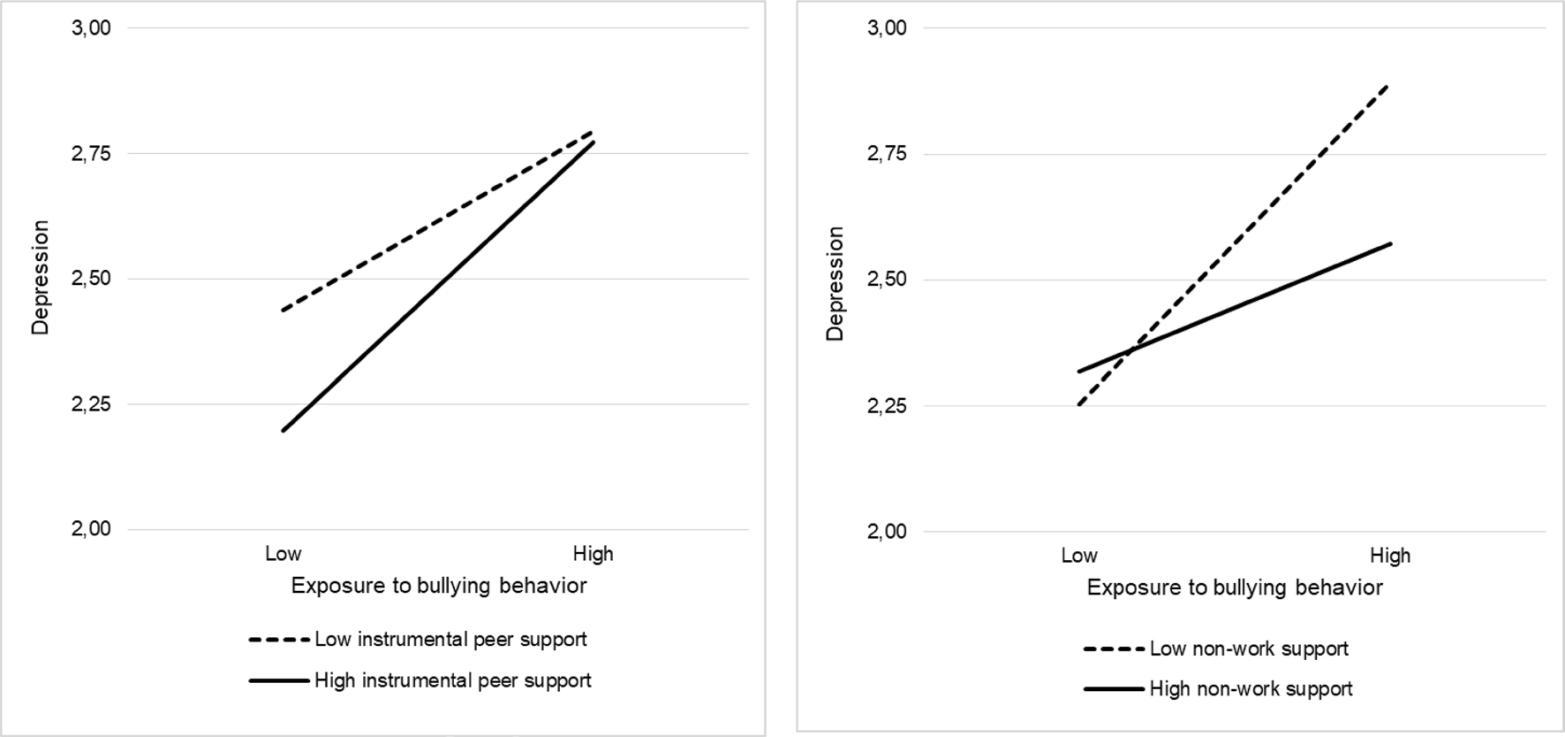
Interaction between bullying behavior and instrumental peer support (left) and non-work support (right), with depression as dependent variable. Low and high values are based on the 16th and 84th percentile, respectively

To address our research questions concerning the buffering effects of different forms of support, we consider their effects on each outcome in turn. Starting with depression, we found that both emotional peer support (*b* = − 0.242, SE = 0.047, *t* = 5.149, *p* < 0.001) and company support (*b* = − 0.108, SE = 0.033, *t* = 3.273, *p* < 0.01) had significant negative effects. Initial analyses (not shown), where we included each interaction term separately, showed significant interactions between exposure to bullying behavior and instrumental peer support (*b* = 0.112, SE = 0.039, *t* = 2.872, *p* < 0.01), emotional peer support (*b* = 0.079, SE = 0.030, *t* = 2.633, *p* < 0.01) and company support (*b* = 0.084, SE = 0.030, *t* = 2.800, *p* < 0.01). The nature of the interactions was similar: all three forms of support buffered the negative impact of exposure to bullying especially for those experiencing low levels of bullying behavior. When all four interaction terms were included in the model simultaneously (Table 3), the interactions of exposure to bullying behavior with instrumental peer support (*b* = 0.137, SE = 0.068, *t* = 2.015, *p* < 0.05) and with non-work support (*b* = − 0.144, SE = 0.049, *t* = 2.939, *p* < 0.01) were significant. As depicted in Fig. 1, instrumental peer support buffered the negative effect of exposure to bullying especially for those experiencing low levels of bullying. By contrast, non-work support reduced the negative effect of bullying especially for those exposed to high levels of bullying.

Turning to anxiety as dependent variable, we found that of the four forms of support, only company support had a significant negative effect (*b* = − 0.150, SE = 0.041, *t* = 3.659, *p* < 0.001). Initial analyses (not shown), adding each interaction term separately, showed significant interactions of exposure to bullying behavior with instrumental peer support (*b* = 0.101, SE = 0.049, *t* = 2.061, *p* < 0.05) and with company support (*b* = 0.095, SE = 0.038, *t* = 2.500, *p* < 0.05). Both instrumental peer support and company support buffered the negative impact of bullying especially for those experiencing low levels of bullying. When all four interaction terms were included in the model simultaneously (Table 3), none of the interaction terms were significant.

**Fig. 2:**
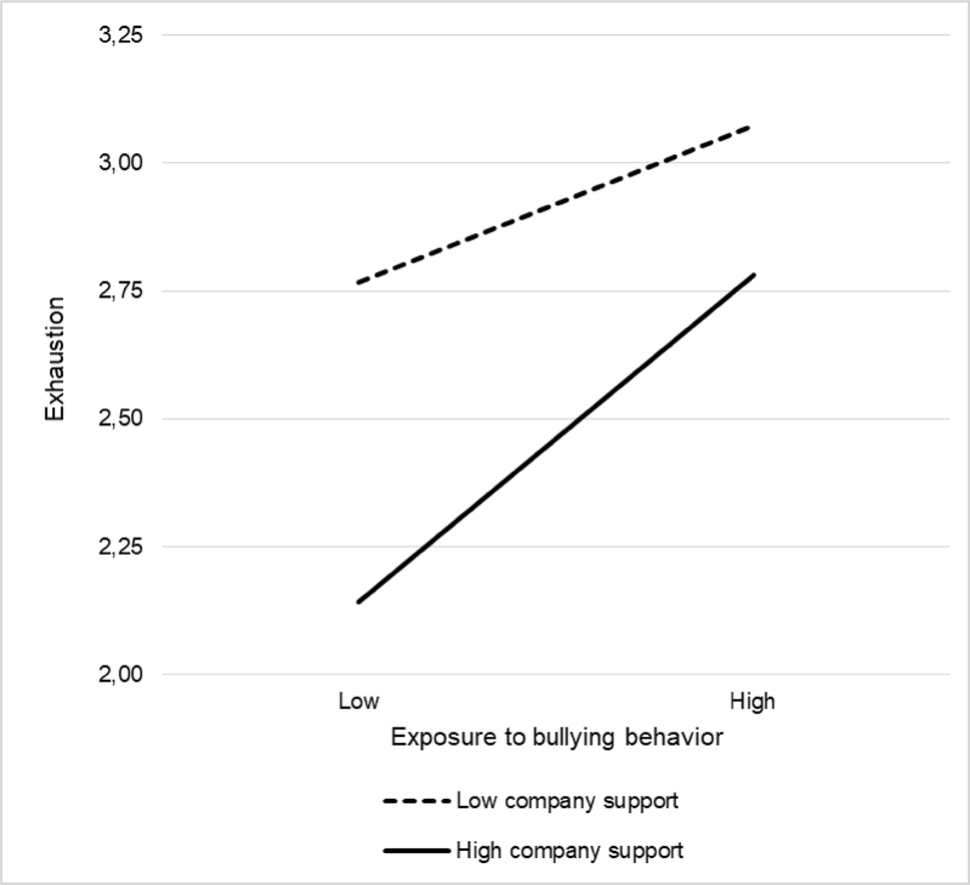
Interaction between bullying behavior and company support with exhaustion as dependent variable. Low and high values are based on the 16th and 84th percentile, respectively

As shown in Table 3, exhaustion was significantly lower for those who reported higher emotional peer support (*b* = −0.150, SE = 0.049, *t* = 3.061, *p* < 0.01) and company support (*b* = − 0.154, SE = 0.034, *t* = 4.529, *p* < 0.001). When adding each interaction term separately (models not shown), we found significant interactions between exposure to bullying behavior and instrumental peer support (*b* = 0.145, SE = 0.039, *t* = 3.718, *p* < 0.001), emotional peer support (*b* = 0.123, SE = 0.036, *t* = 3.417, *p* < 0.001) and company support (*b* = 0.144, SE = 0.033, *t* = 4.364, *p* < 0.001). All three forms of support buffered the negative impact of bullying especially for those experiencing low levels of bullying. When including all four interaction terms simultaneously (Table 3), only the interaction between exposure to bullying behavior and company support remained significant (*b* = 0.124, SE = 0.058, *t* = 2.138, *p* < 0.05). The nature of the interaction was unchanged, i.e. company support reduced the negative impact of bullying especially for those exposed to low levels of bullying (Fig. 2).

## Discussion

This study investigated the effectiveness of four forms of support in buffering the negative impact of exposure to bullying behaviors on depression, anxiety and exhaustion among seafarers on international commercial vessels. We found that all forms of social support reduced depression and exhaustion, and all but non-work support reduced anxiety. However, only some forms of support buffered the negative impact of exposure to workplace bullying, and they varied with regard to the outcomes and the nature of the buffering effect.

While these findings are broadly in line with the buffer theory of social support (Cohen and Wills 1985), our study makes two contributions that add to previous research. First, previous studies have usually examined one interaction at a time, often taking a broad brush approach merging different outcomes and/or different forms of support in their measures. While some studies have already drawn attention to the need to distinguish between support from different sources (e.g. Blomberg and Rosander 2020; Nielsen et al. 2020a), our study adds to this by showing that the buffering effect of social support depends not only on the source of support (e.g. peers, company, non-work), but also on the type of support (e.g. instrumental, emotional) and on the outcome considered (e.g. depression, anxiety, exhaustion). To move forward, this highlights the need for a more nuanced approach that differentiates between the moderating effects of different forms of support and for different outcomes. From a practical perspective, this will be important for identifying effective ways of supporting targets of bullying.

Second, examining the visualizations of the interaction effects, we found that the nature of the interactions differed, i.e. different forms of support were effective in buffering the impact of low vs. high levels of bullying behavior. While previous studies have described the interactions identified, the nature of these interactions and potential patterns in their occurrence have received little attention. A notable exception, in their review of workplace bullying research, Nielsen and Einarsen (2018) note that the individual and contextual moderators studied to date tend to buffer the impact of bullying at low but not at high levels of bullying. Extending this, our findings suggest that differences in the nature of the interaction might be related to the form of support considered. Instrumental peer support and company support (primarily instrumental) were most effective at buffering low levels of bullying behavior. By contrast, non-work support (primarily emotional) mitigated high levels of bullying behavior. This suggests that different forms of support may be effective at different levels of bullying behavior.

A possible explanation for this could be that the severity of bullying influences targets’ and bystanders’ interpretation of the situation (Huitsing et al. 2012), and hence their reactions. First, low levels of negative behavior might not be interpreted as “bullying”, but might instead be considered what Einarsen et al. (2009) label “occasional negative encounters”. Targets may attribute such behavior to a stressful work environment or occupational culture rather than perceive it as directed at them personally (Huitsing et al. 2012; Kitada 2010). In this situation, in line with the trauma perspective, targets may find reassurance in being part of ongoing supportive interactions on board (e.g. receiving instrumental support with a work task) or in working for a company that can be relied upon to care for its employees (e.g. receiving timely support with practical matters).

By contrast, high levels of negative behavior may be more easily interpreted as actual “bullying”. In this situation, instrumental support from crew members and the shipping company may be insufficient to reassure targets and mitigate the impact of severe negative behavior. In a seafaring context with an occupational culture that values “toughness”, requesting, receiving and providing support may be difficult, especially with regard to interpersonal and/or emotional matters. In addition, in a context where employees may have to work and live together for months at a time, bystanders may be hesitant to become associated with a target of bullying, fearing negative repercussions for themselves. Such reluctance may be especially strong when the perpetrator is a high-ranking officer (Kitada 2010).

Instead, non-work support might become essential at high levels of bullying behavior. In general, seafarers tend not to share problems concerning their life on board with family and friends (Kitada 2010; Tang 2009), well aware that discussing their experiences with them will not solve their problems, but only make their family worry. This may explain the non-significant main effects of non-work support in our study. However, when pressure is high, family and friends may be a last resort. In line with the trauma perspective, communication with family members may remind seafarers of their role as valued family members and providers, thus reinforcing their sense of purpose and meaning of life. As indicated by our findings, this may be especially important in reducing the negative impact of bullying on depression.

Considering the buffering effects of different forms of support at different levels of bullying behavior, it is possible that there might be a temporal dimension. That is, as described in the interview study by D’Cruz and Noronha (2011), the impact of bullying and the buffering effect of different forms of social support may unfold over time. We would, therefore, encourage future studies to take a longitudinal approach to explore these processes, and compare the roles of instrumental and emotional support from different sources, and for different outcomes.

Comparing our findings with previous studies also raises questions about the role of context. Our study examined the role of social support specifically among seafarers. Based on the similarities with workers in other male-dominated, hazardous and remote occupations (e.g. offshore oil and gas industry, deep-sea fishing, transnational construction work), we expect similarities regarding the buffering effects of different forms of social support. However, the effects of social support may not generalize to other types of contexts. For instance, we found that non-work support buffered the impact of bullying on depression. By contrast, in a large-scale study of employees in 96 organizations, mostly engaged in office, service or sales work, non-work support did *not* buffer the negative impact of bullying (Nielsen et al. 2020a). This difference could be due to differences in the measurement or the outcomes examined, but it could also indicate the possibility that the buffering effect of social support may be context-dependent. While context-specific mitigating factors may have high practical relevance for those concerned, they are easily overlooked when the research focus is on sample averages. Therefore future studies should pay close attention to organizational and occupational contexts.

## Limitations and directions for future research

Similar to previous studies that have examined social support as a buffer of the negative impact of bullying on employee well-being (e.g. Blomberg and Rosander 2020; Gardner et al. 2013), our study is based on a cross-sectional survey using self-report measures.

The cross-sectional design does not allow definitive conclusions about the direction of causality. Although theoretical work and longitudinal studies generally support the idea that workplace bullying influences physical and mental health, the opposite direction of influence cannot be ruled out (Boudrias et al., 2021). Further, in this study we followed previous theoretical arguments (Cohen and Wills 1985) in considering social support as a buffer. However, an alternative interpretation, with social support not as a buffer but as an outcome (i.e. high levels of distress might prompt targets to seek support), cannot be ruled out. While to our knowledge this has not been tested in previous studies, it might explain reverse buffering effects, such as the association of family support with high levels of co-worker incivility and depression in a study by Lim and Lee (2011). Replications using longitudinal designs would also be desirable to investigate the impact of bullying and the buffering effect of social support over time.

While self-report data may increase common method bias (Podsakoff et al. 2003), this is of less concern in our study where the main interest is in interactions, as significant results cannot be due to common method bias (Siemsen et al. 2010). Moreover, the differences in the effects of the four forms of support for the three outcomes are difficult to explain by common method bias.

The measures for exposure to bullying, depression and anxiety were based on validated measures, but the time frame was adapted to refer to the last seven days rather than the last two weeks (PHQ-2, Kroenke et al. 2003; GAD-2, Kroenke et al. 2007) or the last 6 months (S-NAQ; Escartin et al. 2019; Notelaers et al. 2019). This reduces direct comparability with previous studies using these scales. However, in the context of this study, a longer time frame would have entailed a choice between excluding seafarers with shorter time on board at the time of the survey (hence biasing the sample), or asking seafarers with shorter time on board to report about a shorter time frame (hence reducing comparability among respondents’ reports) or asking them to report about experiences on board as well as experiences ashore that fell within the time frame (hence reducing comparability). To ensure comparability among the answers by our respondents, we therefore chose a seven day time frame.

As we used the S-NAQ scale, a validated measure of exposure to workplace bullying (Notelaers et al. 2019), another limitation of our study is that this measure does not provide information about the identity of the perpetrator. As noted by Blomberg and Rosander (2020, p. 487), it is possible that the relative hierarchical position of target and perpetrator (e.g. being bullied by a supervisor vs. being bullied by a co-worker) may affect what type of social support is most effective for targets. Further, the interpretation of negative behavior and the perceived risk associated with soliciting and providing support may play a role as well. Such perceptions may be shaped by the occupational and organizational context. Therefore, replicating the study in different occupational and organizational contexts, including remote and non-remote occupations would be desirable.

## Conclusion and practical implications

Our findings showed that targets of workplace bullying report significantly higher levels of depression, anxiety and exhaustion. Although more research will be needed to better understand how and why different forms of support differ in their effects on different outcomes, the general pattern suggests that social support had beneficial effects for seafarers’ well-being by improving well-being and/or by reducing the negative impact of bullying.

For seafarers, support from colleagues in the immediate work environment reduced depression and exhaustion directly (emotional peer support) and reduced the negative impact of bullying on depression (instrumental peer support). Hence, interventions should aim at encouraging the development of peer support on board, for instance by offering permanent (rather than fixed term) contracts, allowing sufficient time for rest and informal interactions, and by ensuring that crew members are fluent in the vessel’s working language (Sampson 2013).

In addition, the importance of company and non-work support must not be overlooked. Company support, in particular, contributed to lower depression, anxiety and exhaustion, and mitigated the negative impact of low levels of bullying behavior on exhaustion. By contrast, non-work support may take on special importance at high levels of bullying behavior. While the ability to communicate with family and friends may be taken for granted among shore-based employees, this is not so for seafarers (Sampson et al. 2018) and workers in similar remote occupations. Hence, access to affordable, fast and reliable Internet or other means of communication is essential, and should be a focus of interventions. Moreover, because seafarers may be hesitant to share negative experiences with colleagues, company representatives or even with family and friends, access to independent professional support may be an important alternative.

Because social support can reduce but not completely eliminate the negative impact of bullying, interventions should also aim at preventing or reducing workplace bullying. Previous studies have shown that the work environment and general psychosocial conditions play an important role in the onset of bullying (Salin and Hoel 2020). As such, companies can do much to prevent bullying behavior by creating a work environment where employees feel safe and valued, and take prompt action to stop bullying and support targets (Piñeiro and Kitada 2020; Salin 2013). Providing information on bullying as well as providing training on constructive conflict management for crew members on all levels, thereby also reaching out to potential perpetrators of bullying, can further contribute to reducing the risk of bullying arising in the first place.

## Data Availability

The datasets generated and/or analyzed during the current study are available from the corresponding author on reasonable request.

## Footnotes

1 Because we measured exposure during the past seven days rather than during the past six months, we use the term “bullying behavior” rather than “bullying”. To label something as bullying, definitions would typically require the behavior to be systematic and long-term (Einarsen et al. 2020).
